# Supplementary material for: A Global Screen for Assembly State Changes of the Mitotic Proteome by SEC-SWATH-MS
Source: Cell Syst. 2020 Feb 26;10(2):133–155.e6. doi: 10.1016/j.cels.2020.01.001 (PMC7042714; doi:10.1016/j.cels.2020.01.001)

Q9NZZ3 | CHMP5_HUMAN | CHMP5 C9orf83 SNF7DC2 CGI−34 HSPC177 PNAS−114 PNAS−2
Monomer MW [kDa]: 24.571 Monomer expected elution fraction: 52

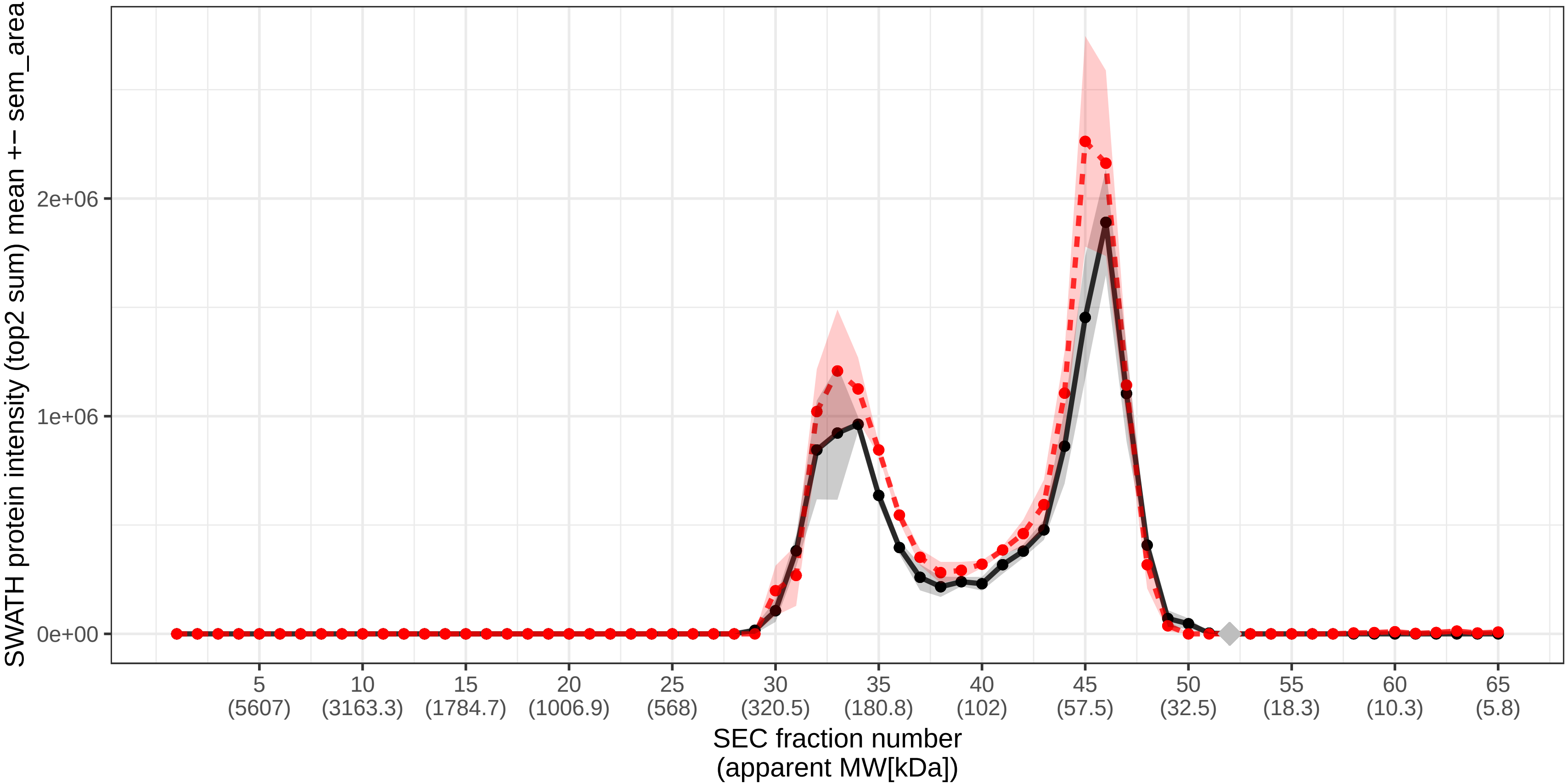

Supplement: Data S1. SEC-SWATH-MS Protein Chromatograms, Related to Figure 1 [file mmc6.zip › SECchrom_Q9NZZ3_CHMP5_HUMAN_CHMP5_C9orf83_SNF7DC2_CGI-34_HS.pdf]
